# Effects of Insecticide and Herbicides on Thyroid Disturbances in Zebrafish

**DOI:** 10.3390/toxics12080570

**Published:** 2024-08-03

**Authors:** Tingting Ma, Xiangji An, Peng Wu, Xiaoli He, Yongming Luo

**Affiliations:** 1 College of Resource Environment & Tourism, Hubei University of Arts & Science, Xiangyang 441053, China; ttmaxiaotu@126.com (T.M.); axj140235@outlook.com (X.A.); 2 Key Laboratory of Soil and Sustainable Agriculture, Chinese Academy of Sciences, Nanjing 211135, China; ymluo@yic.ac.cn; 3 Hubei Key Laboratory of Low Dimensional Optoelectronic Materials & Devices, Hubei University of Arts & Science, Xiangyang 441053, China; 4 Jiangsu Rainfine Environmental Science & Technology Co. Ltd., Nanjing 210009, China; woopon@163.com

**Keywords:** zebrafish, thyroid dysfunction, environmental endocrine disruptors, life stage, hypothalamic–pituitary–thyroid axis

## Abstract

Thyroid cancer usually begins with thyroid dysfunction and nodules and has become the most common cancer globally, especially in women. Although the causes of thyroid dysfunction are complex, the presence of environmental pollutants, especially certain pesticides as established mutagens, has been widely accepted. Zebrafish (*Danio rerio*) have similar toxic reactions and signal transduction pathways to humans and are very similar to humans in physiology, development, and metabolic function. Here, the direct toxicity effects and mechanisms of different insecticides and herbicides on zebrafish thyroid functions and indirect toxicity effects originating from thyroid dysfunction were summarized and compared. The overall toxicity of insecticides on the zebrafish thyroid was greater than that of herbicides based on effective concentrations. Penpropathrin and atrazine were more typical thyroid disruptors than other pesticides. Meanwhile, chiral pesticides showed more sophisticated single/combined toxicity effects on both parental and offspring zebrafish. Besides thyroid hormone levels and HPT axis-related gene expression alteration, developmental toxicity, immunotoxicity, and oxidative damage effects were all observed. These data are necessary for understanding the thyroid interference effect of pesticides on humans and for screening for thyroid disruptors in surface water with zebrafish models for the pre-assessment of human health risks and ecological risk control in the future.

## 1. Introduction

The thyroid gland is the largest endocrine gland in the human body. Thyroid dysfunction directly leads to abnormal cardiac blood output, lipid metabolism disorders, atherosclerosis, poor bone function, abnormal kidney function, and even infertility [1,2,3,4]. Thyroid cancer—the leading endocrine tumor with a rising global incidence, especially in women—is largely influenced by both genetic and environmental factors, in addition to nutritional conditions and lifestyle habits [5]. Dysregulation of thyroid hormone (TH) levels—key regulators of energy metabolism—can severely perturb physiological growth, metabolism, differentiation, and homeostasis in adults and developmental processes in utero, even when TH levels are within the normal range [6,7]. Toxicological and human studies suggest that some pesticides act as endocrine disruptors with effects on many systems, especially associations between some insecticides/herbicides and thyroid-related alterations, including modulation of the synthesis, transportation, and metabolism of thyroid hormones, interference with hormone receptors, and modulation of related gene expression [8]. In 2020, the average annual use of pesticides in China was second only to that of the United States and Brazil, ranking third in the world, with 26.96 and 41.49% of pesticides and herbicides, respectively [9]. After 100 years of development, the world pesticide industry has annual sales of USD ~70 billion and has commercialized more than 1500 pesticide products and ~30,000 preparation products [10]. However, not all insecticides and herbicides are classified as endocrine disruptors, and some do not have the same effect on thyroid function or have the same mechanism of action [11].

Zebrafish (*Danio rerio*) are the most commonly used model organisms in toxicology studies, filling the gap between in vitro and mammalian medical models with considerable advantages. As a small freshwater species, zebrafish offer many advantages for the study of organ and tissue development that are not provided by other model systems [12]. Among vertebrate model organisms, zebrafish are superbly suited for the rapid generation of sequence-targeted mutant lines, characterization of phenotypes, including gene expression patterns, and generation of human disease models [13]. Some zebrafish phenotypes resemble human disease states, and mapping and sequencing data have revealed zebrafish genes with notable homology to human disease-causing genes [12]. New efforts in the zebrafish community to develop models of adult diseases that range from cancer to the heart, infectious and age-related diseases, and those that relate to toxicology and complex social behaviors have excited the scientific world [14].

**Figure 1:**
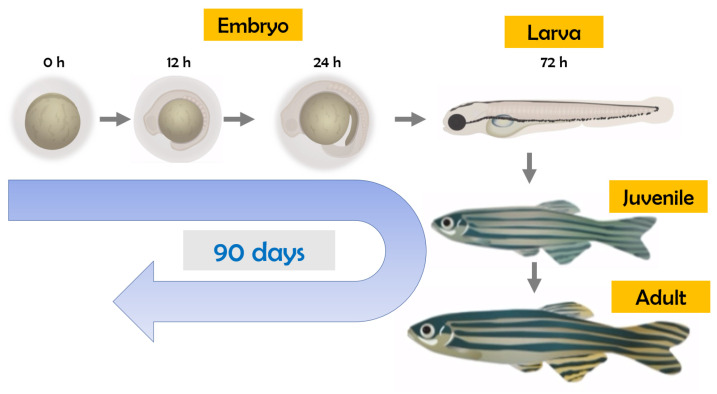
Life cycle of zebrafish (*Danio rerio*).

As well-known endocrine-disrupting chemicals, insecticides/herbicides may act on the hypothalamic–pituitary–gonadal (HPG), hypothalamic–pituitary–thyroid (HPT), and hypothalamic–pituitary–adrenocortical (HPA) systems [15]. For thyroid disruption studies using zebrafish, observations at multiple life stages are important (Figure 1), considering the dynamic nature of the development and regulatory feedback control of the thyroid endocrine system during the embryo–larval period up to the adult stage [16].

For a long time, pesticides have been widely considered as suspected endocrine disruptors, but the endocrine-disrupting effects of different pesticide types, such as insecticides and herbicides, especially the differences in thyroid-disrupting effects, effective concentrations, and mechanisms, have not been specified. Here, considering the absolute proportion of pesticides and herbicides in total pesticide use, we summarized and compared the main toxic effects and mechanisms of different types of insecticides and herbicides on thyroid function and other related systems in zebrafish at different life stages. Clarifying the intergenerational toxicity and mechanisms of pesticide thyroid interference on parental and offspring zebrafish is an important reference framework for high-throughput screening of thyroid disturbance pollutants in water and bioassay measures for the early warning and monitoring of water pollution, pre-assessment of combined water pollution risk, and watershed pollution control in the future.

## 2. Materials and Methods

Comprehensive electronic databases were retrieved for literature up till May 2024. An electronic literature search of four English databases (Web of Science, Science Direct, Springer Link, and Wiley) and three Chinese databases (CNKI, VIP, and WANFANG DATA) was conducted. Studies that examined insecticide or herbicide, pesticide but not fungicide, zebrafish, thyroid hormone, and hypothalamic–pituitary–thyroid axis were included. Master’s and doctoral dissertations were also included. In the “Identification” process, 52 English references and 23 Chinese references were collected. After “Screening” for repetition elimination, trial data reliability confirmation (quality assurance/quality control, QA/QC), and so on, 29 English references and 13 Chinese references were included. The QA/QC of the toxicity testing in the literature was confirmed by checking negative/positive control, numbers of parallel samples and methods, and results of data statistical analysis.

## 3. Target Pollutant Effects

### 3.1. Thyroid Disturbance Effects of Insecticides

Insecticides are chemical agents used to control pests. According to the chemical structure, insecticides can be classified as pyrethroid, organophosphorus, organochlorine, neonicotinoid, carbamate, acaricide, insect growth regulator, silkworm toxin, benzoyl urea, plant, and microbial insecticides, among others [17]. Insecticides, which can only kill pests but are not suitable for preventing and curing diseases, have been applied in large amounts and are the most varied among all pesticide types. Insecticides play a very important role in increasing agricultural production and solving human food problems, but they also cause notable environmental pollution [18]. Most insecticides are stable and termed persistent organic pollutants that easily accumulate in soil and organisms [19]. Controlling pests is closely related to the reduction or even extinction of natural pest enemies and other beneficial organisms [20]. As a suspected thyroid function disruptor in zebrafish, relevant studies have been carried out on insecticides; the relevant research concentrations, exposure times of target pollutants, and test life stages are listed in Table 1. Direct thyroid interference and indirect toxic effects on related systems caused by insecticides are listed in Table S1. Fenpropathrin is the most serious thyroid function disruptor among all the investigated pesticides, with an effective concentration of 0.015 µg/L. Bifenthrin, permethrin, and λ-cyhalothrin are the second most toxic thyroid-disruptive insecticides with effective concentrations of 0.1 µg/L (Table 1).

#### 3.1.1. Effects of Insecticides on Thyroid Directly Related Indices

Thyroid hormones play crucial roles in vertebrate development, growth, metabolism, and reproduction [21]. Thyroxine (T4) and 3,5,3′-triiodothyronine (T3) are released from the thyroid into the peripheral blood and function by binding to thyroid hormone-binding proteins for transport. Disruption of the TH balance can be directly observed from alterations in T4 and T3 levels. Increased T3 levels were observed in F0 embryo/larvae/adult zebrafish treated by insecticides including acaricide (azocyclotin) [22], neonicotinoid (imidacloprid) [23], organophosphorus (chlorpyrifos) [24,25,26], (triazophos) [23], and pyrethroids (bifenthrin [27,28], permethrin [27,29], and λ-cyhalothrin [27]). T4 levels increased when exposed to the carbamate pesticide, carbofuran, at a concentration of 50 μg/L for 4 h [30] and the pyrethroid insecticides (permethrin [27,29] and λ-cyhalothrin [27]). However, T4 levels decreased when singly exposed to imidacloprid and triazophos [23]. In F0 zebrafish treated with bifenthrin, both increased [28] and decreased [27] T4 levels were observed. Hypothyroidism was evident in ethylene thiourea-exposed larvae, which showed a reduced number of follicles [31]. Thyroid and TH signaling were affected in second-generation larvae even when not directly exposed, suggesting a parental exposure role [31]. Fipronil, a phenylpyrazole insecticide, decreased T3 and T4 levels in both the F0 and F1 generations, particularly in F1 [32]. However, in the F1 generation of ethylene thiourea-exposed zebrafish, increases in both T3 and T4 levels were detected [31]. The conversion from T4 to T3 directly resulted in a rise of the T3/T4 ratio, indicating a breaking of the balance of free T3/free T4, observed in treatments with azocyclotin [22], bifenthrin [27], permethrin [27,29], and λ-cyhalothrin [27].

Deiodination is a critical process by which a minimally active T4 molecule is converted into a favorable ligand for the TH receptor, T3 [33]. T3 binds to thyroid receptors and mediates their action in target organs. According to deiodination characteristics and distribution, iodothyronine deiodinases type 1, 2, and 3 (Dio1, Dio2, and Dio3) function in tissue-specific regulation of TH bioavailability via a potent mechanism of TH activation (Dio1 and Dio2) or inactivation (Dio3). In most cases, insecticides induced the upregulation of Dio2, including pyriproxyfen [34], chlorpyrifos [24], bifenthrin [27,28,35], permethrin [21,27,29,36], β-cypermethrin [21], λ-cyhalothrin [27,35,36], and ethylene thiourea [26]. However, Dio2 is downregulated upon exposure to azocyclotin [22], imidacloprid, and triazophos [23]. Overall, a notable increase in Dio2 levels in zebrafish supports the result of an increased T3 to T4 ratio [37]. Suppressing Dio2 expression might be a possible mechanism by which insecticides disrupt the thyroidal system in zebrafish [22]. Meanwhile, Dio1 is upregulated by pyriproxyfen [34], chlorpyrifos [24], bifenthrin [35], permethrin [27,29,36], and λ-cyhalothrin [27,35,36] and down-regulated by a single exposure to imidacloprid and triazophos [23].

The enantioselectivity of chiral pesticides may cause gene expression changes during developmental toxicity in zebrafish embryos and larvae. For example, Rac-metalaxyl only upregulates Dio2, whereas R-metalaxyl suppresses Dio1 expression and induces Dio2 expression [38]. Ethiprole and flufiprole were developed as alternatives to fipronil. S-(-)-flufiprole and S-(-)-ethiprole showed anti-thyroid hormone effects by increasing the expression of Dio1 [39,40]. S-fenpropathrin showed a stronger thyroid-disrupting effect than R-fenpropathrin [41]. There was a notable thyroid-disrupting effect of fenpropathrin in zebrafish by downregulating the transcription of Dio2 genes in the juvenile stage, but at the embryo and yolk-sac larval life stages, this occurred mainly by downregulating the transcription of Dio1 genes [41]. Similar transcription could also be recognized between generations, such as the stimulation of Dio1 and Dio2 transcription in both F0 and F1 zebrafish [32] and the upregulation of Dio3b in F0 and F1 caused by chlorpyrifos [42] and ethylene thiourea [31], respectively. An increase in free T3 levels/signaling was confirmed by the transcriptional regulation of deiodinases and T3-regulated mRNAs (cpt1, igf3, and insulin growth factor binding protein 1a gene/igfbp1a) [26,31].

Generally, the thyroid endocrine system in fish is primarily controlled by the HPT axis, which regulates TH synthesis, secretion, transport, and metabolism to maintain TH homeostasis [29]. Gene transcription along the HPT axis is also markedly affected by insecticide exposure [32]. Thyroid-stimulating hormone (TSH), encoded by tshba, is the most important thyroid function regulator [25]. It is a hormone secreted by the pituitary gland that excites the follicular cells of the thyroid gland and influences and controls the entire sequence of reactions that form T3 and T4. Pyriproxyfen [34], imidacloprid [23], and triazophos [23], showed anti-thyroid hormone effects by reducing the expression of TSHβ as well as Rac-fenpropathrin and R-fenpropathrin in juvenile zebrafish [41]. Up-regulated TSHR and/or TSHβ expression after treatment with bifenthrin [27,28,35], permethrin [21,27,29,36], β-cypermethrin [21], λ-cyhalothrin [35,36], chlorpyrifos [24,25], fipronil [32], S-(-)-flufiprole, and S-(-)-ethiprole [39,40] in the F0 generation and TSHβ expression after treatment with ethylene thiourea in the F1 generation [31] were also observed. Corticotropin-releasing hormone (CRH), secreted by the hypothalamus, stimulates the release of TSH from the pituitary gland, which coordinates the synthesis and release of TH [43]. Except when treated with Rac-fenpropathrin or S-fenpropathrin [41], CRH expression is up-regulated with chlorpyrifos [24,25], fipronil [32], bifenthrin [27,28,35], permethrin [27,29,36], and λ-cyhalothrin [35,36].

The sodium-iodide symporter (NIS) is an intrinsic plasma membrane protein that mediates the active transport of iodide in the thyroid gland and several other extra-thyroidal tissues [44]. Except for permethrin [36,45], NIS transcription is promoted when exposed to R-metalaxyl [38], bifenthrin [35], Rac-fenpropathrin, S-fenpropathrin [41], λ-cyhalothrin [35,36,45], and fipronil in both F0 and F1 [32]. NIS, encoded by slc5a5, which is notably upregulated by chlorpyrifos [25], is involved in thyroid hormonogenesis [46]. Thyroglobulin (TG) is a macromolecular glycoprotein secreted by thyroid follicular epithelial cells, most of which is synthesized by thyroid cells and released into the residual cavity of thyroid follicles. TG is involved in TH synthesis and TSH, thyroid iodine deficiency, and thyroid-stimulating immunoglobulins can stimulate its production. For example, in zebrafish treated with fipronil [32], bifenthrin [35], permethrin [36], λ-cyhalothrin [35,36], and ethylene thiourea [31], the increased TSH stimulates TG production. However, inhibition of TG production was observed with chlorpyrifos treatments, even with increased TSH levels [24].

**Table 1:** Parameters on thyroid disturbance effects of insecticides on zebrafish [21,22,23,24,25,26,27,28,29,30,31,32,33,34,35,36,37,38,39,40,41,42].

Insecticide Type	Name	MC. ^1^	EC. ^2^	Time	Life Stage	Refs.
(1) Acaricide	Azocyclotin	0.36 µg/L	0.24 µg/L	72 hpf ^3^	Embryo, larvae	[22]
(2) Benzoyl urea	Metalaxyl	200 mg/L	100 mg/L	96 hpf	Embryo, larvae	[38]
(3) Carbamate	Carbofuran	500 µg/L	50 µg/L	4 h	Adult	[30]
(4) Insect growth regulator	Pyriproxyfen	250.7 μg/L	111.7 μg/L	33 d	Larvae	[34]
(5) Neonicotinoid	Imidacloprid	6.08 mg/L	0.38 mg/L	96 h	Whole life	[23]
(6) Organophosphorus	Chlorpyrifos	106.7 μg/L	26.7 μg/L	96 h	Larvae	[24]
		65 μg/L	13 μg/L	10 dpf ^4^	Larvae	[25]
		300 nM	30 nM	180 dpf	Larvae	[26]
		3 mg/L	0.75 mg/L	96 hpf	Embryo, larvae	[42]
	Triazophos	0.032 mg/L	2 µg/L	96 h	Whole life	[23]
(7) Phenylpyrazole	Ethiprole	20 mg/L	1.278 mg/L	10 d	Embryo	[40]
	Fipronil	10.0 µg/L	1.0 µg/L	28 d	F0 adult, F1 embryo	[32]
	Flufiprole	20 mg/L	1.399 mg/L	10 d	Embryo, larvae	[40]
(8) Pyrethroids	Bifenthrin	10 μg/L	3 μg/L	72 hpf	Embryo, larvae	[27]
	Bifenthrin	60 μg/L	0.1 μg/L	120 h	Embryo	[35]
	Bifenthrin	3 μg/L	1 μg/L	14 d	Embryo, larvae	[28]
	Fenpropathrin	15 μg/L	0.015 μg/L	144 h	Embryo, larvae, Juvenile	[41]
	Fenvalerate	500 nM	2.5 nM	96 hpf	Embryo	[36]
	Fenvalerate	500 nM	2.5 nM	96 hpf	Embryo	[45]
	Permethrin	10 μg/L	1 μg/L	72 hpf	Embryo, larvae	[27]
	Permethrin	500 nM	0.1 μg/L	168 h	Embryo, adult	[36]
	Permethrin	10 μg/L	1 μg/L	72 hpf	Larvae	[29]
	Permethrin	500 nM	2.5 nM	96 hpf	Embryo	[45]
	Permethrin	0.75 μM	0.5 μM	72 h	Embryo, larvae	[21]
	β-cypermethrin	0.75 μM	0.25 μM	72 h	Embryo, larvae	[21]
	λ-cyhalothrin	10 μg/L	1 μg/L	72 hpf	Embryo, larvae	[27]
	λ-cyhalothrin	10 μg/L	1 μg/L	120 h	Embryo	[35]
	λ-cyhalothrin	500 nM	0.1 µg/L	168 h	Embryo, adult	[36]
	λ-cyhalothrin	500 nM	25 nM	96 hpf	Embryo	[45]
(9) Silkworm toxin	Ethylene thiourea	100 μM	100 μM	180 dpf	F1 larvae, adult, F2 embryo, larvae	[31]
		100 µM	100 µM	180 dpf	Larvae, adult	[26]

^1^ MC, Maximum concentration of the toxicology test; ^2^ EC, effective concentration of thyroid interference effects; ^3^ hfp, hours post fertilization; ^4^ dpf, days post fertilization.

Among chiral enantiomers, Rac-fenpropathrin and S-fenpropathrin inhibit TG in embryos and juveniles, whereas R-fenpropathrin stimulates TG in larval zebrafish [41]. For TH synthesis, the enzyme thyroid peroxidase (TPO) controls the organization of iodine and transfers it to TG [46]. While R-metalaxyl and λ-cyhalothrin [36] suppress TPO expression [38], F0 zebrafish treated with chlorpyrifos [25], permethrin [36], and ethylene thiourea [31], and fipronil-treated F0 and F1 show elevated TPO expression. Based on the enantioselectivity of chiral pesticides, Rac-fenpropathrin reduces TPO in larvae, whereas S-fenpropathrin increases it in embryos [41]. Transthyretin (TTR) is also involved in TH metabolism. As an important TH transport protein, TTR, which binds and transports THs to target tissues, plays a crucial role in the thyroid axis in fish. TTR upregulation is induced by chlorpyrifos [25], fipronil [32], bifenthrin [27,35], permethrin [27,29], λ-cyhalothrin [27,35], and S-fenpropathrin [41], but not by R-fenpropathrin in juveniles [41]. UGT1ab upregulation might be responsible for the decrease in T4 when F0 zebrafish are exposed to chlorpyrifos [25], fipronil [32], and bifenthrin [27], particularly when exposed to R-cis-bifenthrin and λ-cyhalothrin [35], and F1 zebrafish exposed to ethylene thiourea [31]. S-fenpropathrin stimulates UGT1ab expression in embryos, whereas Rac-fenpropathrin and R-fenpropathrin inhibit this in larvae and juveniles [41].

TH receptors, TRα and TRβ encoded by THRα and THRβ genes, respectively, are proteins that are prototypically bound to chromatin in the nucleus with a high affinity for DNA recognition sites. When combined with THs, the main function of TH receptors is to transmit information related to development and energy production. Long-term exposure to trace azocyclotin leads to integrated thyroid endocrine disruption in zebrafish by activating thyroid receptor-mediated signaling [22]. In both larvae and juveniles, steady enhancement of TRα and/or TRβ in chlorpyrifos [24,25], triazophos [23], bifenthrin [27,28,35], and β-cypermethrin [21], and a considerable decrease in pyriproxyfen [34], S-(-)-flufiprolem, S-(-)-ethiprole [39,40], fenvalerate [36,45], S-fenpropathrin, and Rac-fenpropathrin, have been shown. TRα and/or TRβ are not consistently changed when zebrafish are exposed to permethrin [21,27,29,36,45] and λ-cyhalothrin [35,36,45] and between generations to fipronil [32].

One of the few genes known to play an essential role in thyroid development is the transcription factor Nkx2.1 [47]. Except for Rac-fenpropathrin and R-fenpropathrin, Nkx2.1 is up-regulated by bifenthrin and λ-cyhalothrin [35] in F0 zebrafish and by fipronil in both F0 and F1 zebrafish [32]. Pax genes are transcription factors that contain a DNA-binding paired domain and are involved in many aspects of organogenesis. Pax8 is involved in thyroid development and is required for late specification or differentiation of follicular cells [47]. Bifenthrin, permethrin, and λ-cyhalothrin [27,29,35] enhanced Pax8 expression, while S-fenpropathrin depressed this, especially in juveniles [41].

#### 3.1.2. Insecticide Effects on HPT Indirectly Related Indices

Disruption of the HPT axis by exogenous compounds affects the normal function of thyroid hormones, ultimately affecting the growth, development, metabolism, and reproduction of fish [41]. The mortality increase when exposed to bifenthrin [28,35], fenvalerate [36,45], permethrin [36,45], and λ-cyhalothrin [35,36,45] can directly reflect the acute toxic effect of target herbicides. For various basic growth indicators, body weight decreases when exposed to azocyclotin [22], pyriproxyfen [34], permethrin [27,29], and λ-cyhalothrin [27] were observed. Besides yolk sac edema caused by metalaxyl [38] and fenpropathrin [41], pericardial edema caused by metalaxyl [38], fenpropathrin [41], permethrin, and β-cypermethrin [21], and heart rate reduction induced by azocyclotin [22], bifenthrin and λ-cyhalothrin [35], are the most commonly detected embryotoxicity. The skeletal development of fish bones is important in determining the growth and locomotion ability of fish because it can support the body of the fish and help them maintain a normal posture and swimming function. The curved spine and short tail, which appear when exposed to metalaxyl [38], bifenthrin [35], fenpropathrin [41], and λ-cyhalothrin [35], could directly affect subsequent predation, avoiding natural enemies, mating, and other behaviors [32,36,45], and even the swim speed of F1 generations [32].

Alterations in gene expression in the antioxidase system, especially the activity of Mn-sod, GPX, Cu/Zn-sod [23], EROD [32], and CAT [23,28], have been activated by imidacloprid, triazophos, fipronil, and bifenthrin. Insecticides induce cellular apoptosis via the caspase pathway, such that both the mRNA expression and activities of caspase3 and caspase9 are notably affected by imidacloprid and triazophos [23]. Upregulation of several apoptosis-related genes, such as bcl-2, ucp-2, and bax by triazophos exposure and p53 by single exposure to imidacloprid and triazophos [23], was observed during zebrafish development. The expression of the immune-related genes CXCL-ClC, CC-chem, IL-1 b, and IL-8 were suppressed, suggesting that the defense function of the immune system in zebrafish was affected by single exposure to permethrin, β-cypermethrin, imidacloprid, and triazophos [21,23]. Estrogen and androgen receptors are widely present nuclear receptors involved in various endocrine systems and oxidative metabolism. Vitellogenin (VTG) is a common biomarker of thyroid dysfunction in zebrafish. The following confirmed the endocrine disruption effects of target pollutants: alteration of AR, ER1, ER2α, and ER2β, caused by single exposure to chlorpyrifos, fenvalerate, permethrin, and λ-cyhalothrin [24,36,45], and alteration of the genes involved in the HPG axis including vtg1, vtg2, cyp19a, and cyp19b, caused by single exposure to chlorpyrifos and metalaxyl [24,38]. Parental exposure to chlorpyrifos could directly affect F1 thyroid and TH signaling and cause even more pronounced sex-dependent damage of hepatic T3 level/signaling in adult female F1 zebrafish [31]. Suppression of hatching and survival rates [22,32,36,38,45] has also been observed for target insecticides.

### 3.2. Thyroid Disturbance Effects of Herbicides

Commercial herbicide formulations are widely used in agriculture to control terrestrial and/or aquatic weeds or harmful plants without affecting the normal growth of crops. Herbicides can be divided into inorganic and organic herbicides. They can enter aquatic ecosystems via drift, runoff, and leaching from the soil or may be applied directly to surface water, resulting in ecological balance disruptions and adverse effects on non-target organisms, including fish [48]. The relevant research concentrations, exposure times to target herbicides, and test life stages are listed in Table 2. Direct thyroid interference and indirect toxicity effects on related systems caused by herbicides are listed in Table S2. For herbicides, the effective concentrations are much higher, at the level of 0.3 µg/L for the most toxic, atrazine, and for the next most effective herbicides, acetochlor and pentachlorophenol, with a concentration of 1 µg/L (Table 2).

#### 3.2.1. Herbicide Effects on Thyroid Directly Related Indices

Increased T4 and T3 levels in zebrafish are caused by butachlor exposure in both early life and adult stages, which vary by gender [49,50,51]. During zebrafish embryo development, pretilachlor upregulates the levels of thyroid hormones T3 and T4 [52]. Strong effects on the thyroid hormone system, especially increased levels of T4, caused the greatest comprehensive endocrine disruption [53]. Glyphosate induces developmental toxicity in fish, possibly due to a strongly decreased T3/T4 ratio [54]. Pentachlorophenol exposure elevated plasma T4 concentrations in male and female zebrafish and depressed T3 concentrations in males only but also in F1 [55]. Exposure to 5.4 mg/L of 2,4-D markedly decreased T4 immunoreactivity and impaired thyroid gland function in zebrafish larvae [56]. Acetochlor showed more complicated effects on TH levels because it could cause endocrine disruption of the thyroid system by lowering the biological activity of T3 and simulating that of T4 in zebrafish larvae [57,58] or by increasing T3 levels and altering T4 levels [28,59,60]. Dio1 [49] and Dio2 [27,28,51,52,59,60,61] were overexpressed at environmentally relevant concentrations of the pesticides acetochlor, butachlor, metolachlor, and pretilachlor. Pentachlorophenol decreases Dio1 expression in both sexes; however, changes in the mRNA levels of Dio2 in the liver are sex-specific [55].

Opposite to the situation in butachlor [49] and glyphosate [54], decreased mRNA expression levels of TSHβ in the brain exposed to pentachlorophenol have been observed. However, both the stimulation and inhibition of TSH or TSHβ could be detected in acetochlor-exposed zebrafish [27,57,62]. Although no direct determination of NIS expression has been made, its coding gene slc5a5 is altered in fish exposed to acetochlor [57,62] and butachlor [49]. The sulfonylurea herbicide ioxynil has a negative effect on thyrocyte development by decreasing the mRNA expression field of both nk2.1a and tg genes in zebrafish [63], while butachlor upregulates the mRNA levels of TG genes [51]. Butachlor upregulated the mRNA levels of CRH genes, while acetochlor downregulated the same mRNA [49,62]. Both acetochlor and butachlor upregulated mRNA levels of the genes involved in TH synthesis [51,57]. The expression of the transport-related protein TTR was enhanced by treatment with butachlor [49,51] and glyphosate [54], but the opposite was observed with acetochlor [57,62]. However, changes in the mRNA levels of TTR genes in the liver are sex-specific [55]. Pentachlorophenol increases liver levels of ugt1ab [55], but a single exposure to acetochlor and butachlor downregulates UGT1ab [49,57,62]. The abnormal expression patterns of HPT axis-related genes trα and trβ have been caused by butachlor [49,51], metolachlor [61], pretilachlor [52], and glyphosate [54]. Alterations in THRA, a gene responsible for proper thyroid function in adult female brain tissue following developmental atrazine exposure, have been confirmed [64]. The increased expression of TRα and TRβ caused by (+)-S-acetochlor [27,60,62] and Rac-acetochlor [62] and changed expression of TRα and TRβ caused by acetochlor [57,62] and (−)-R-acetochlor [27,60,62] cannot be ignored. Changes in the mRNA levels of cytosolic sulfotransferase (sult1 st5) genes in the liver were sex-specific [55] when exposed to pentachlorophenol.

#### 3.2.2. HPT Indirectly Related Indices

In contrast to the toxic effects of insecticides, body weight enhancement has been found with acetochlor [57] and butachlor [49], as well as body length inhibition caused by glyphosate [54]. In addition to yolk sac edema caused by acetochlor [27,57,60,62] and butachlor [27], the coagulation of eggs [27,60] and pericardial edema caused by acetochlor [27,60], butachlor [27], and glyphosate [54], and the reduced heart rate caused by glyphosate [54] are commonly observed. Glyphosate and metolachlor induce swim/gas bladder deficiency in fish as a typical developmental toxicity, likely due to a notably decreased T3/T4 ratio and abnormal expression patterns of HPT axis-related genes [54,61]. Embryotoxicity, including notochord deformation [54,57,62], crooked body [27,60], caudal vertebra deformation [51], and tail, short tail, and even head malformations [54], occurs when exposed to acetochlor, butachlor, and glyphosate. Ioxynil causes a marked reduction in thyroid anlagen size and alters thyroid development in zebrafish [63].

Oxidative stress is a recognized toxic mechanism of herbicides in non-target organisms [54]. The promotion of MDA content [54,57], CAT [28], Cu/Zn-SOD, and GPX [52] activity, and the suppression of SOD and GST activity, and GSH content [49] caused by acetochlor, butachlor, pretilachlor, and glyphosate indicates serious oxidative stress during zebrafish development. Increases in P53 [52,54,61], Bbc3, Cas3 [52], Cas 8, and Cas9 [54] also indicated that cellular apoptosis was induced by herbicides. The stimulation of VTG1, CYP19a, CYP19b, and ERα [27,52] and the inhibition of ERβ1 [52] caused by butachlor and pretilachlor suggest endocrine-disrupting effects. Adverse effects of environmental chemicals on the immune system of aquatic organisms may affect their survival, development, and growth. Alteration of CXCL-CIC, CC-chem, IL-1β, IL-8, iNOS, and TNF-α [27,52,54] induced by acetochlor, butachlor, pretilachlor, and glyphosate clarified the pre-damage to immune systems. Glyphosate-induced premature hatching [54] is quite different from the reduction in the hatching rate [27,57] and even the elevation in mortality [27,28,51] caused by acetochlor and butachlor.

### 3.3. Combined Thyroid Disturbance Effects of Insecticides and Herbicides

Here, because of the complex situation, only pesticide and herbicide combinations were included (Table 3). In the combination of insecticides and herbicides, the thyroid endocrine-disrupting effects in zebrafish can be strengthened compared to single pesticides. All the binary mixtures of bifenthrin and acetochlor markedly changed TH levels of both T3 and T4 and altered gene expressions in the HPT axis such as Dio2, TRα, TSHβ, and CRH have been observed at 0.0072 µmol/L of bifenthrin + 0.004 µmol/L of acetochlor [28,59]. For these two insecticides, the expression of genes related to the HPT axis at the mRNA level revealed that zebrafish embryos were affected by the joint pesticides triazophos and imidacloprid, and greater changes in the expression of Dio1, Dio2, tsh, and vtg1 were observed when exposed to joint pesticides than when exposed to individual pesticides [23]. Free T4 and T3 levels were increased in (ethylene thiourea + chlorpyrifos 30 nM)-F1 larvae, and the expression of T3-targets, igfbp1a and carnitine palmitoyltransferase I (cpt1), were also increased. Whereas other HPT axis-related genes, utg1ab, dio1, and dio3b, were increased in (ethylene thiourea + chlorpyrifos 300 nM)-F1 larvae [31]. For F2 larvae, although not as notable as for F1 larvae, increased free T4 levels and expression of cpt1 and tg in ethylene thiourea + chlorpyrifos 30 nM have also been detected [26,31].

## 4. Discussion

### 4.1. Toxicity Characteristics of Insecticides and Herbicides on Thyroid Dysfunction

#### 4.1.1. Predominant Influencing Factors of Toxic Effects

Pesticides containing chiral carbon, chiral phosphorus, and chiral sulfur atoms with chiral characteristics are termed chiral pesticides. According to the Insecticide/Herbicide/Fungicide Resistance Action Committee, about 30% of chemical pesticides globally have chiral centers, which mainly include pyrethroid and organophosphorus insecticides, amide, aroxyphenoxypropionic ester, and imidazolinone herbicides, and triazole fungicides, among others [60]. In the Chinese pesticide market, the proportion of chiral pesticides has reached 40% and is continuously increasing [61]. Owing to the differences in the spatial structure of their counterparts, chiral pesticides show different and even completely opposing biological effects. For example, the herbicide S-metolachlor shows highly effective herbicidal activity, whereas the enantiomer R-metolachlor mainly exerts mutagenic effects in mammals [62]. Considering the different toxicities of different enantiomers, a thorough understanding of the thyroid-disrupting effects of chiral pesticides should be considered [65]. For example, (+)-S-acetochlor showed stronger thyroid-disrupting effects than (−)-R-acetochlor in TR expression, indicating that enantiomers had different influences on TH secretion, the expression of TH-related key genes, and the binding affinity to thyroid receptors [27,65]. Both S-flufiprole and S-ethiprole exhibited a greater interference effect on gene expression related to the HPT axis in zebrafish than in the R configuration [41]. The toxicity of the two cis-bifenthrin enantiomers in zebrafish embryos was notably enantioselective, and the toxicity of 1R-cis-bifenthrin was stronger than that of 1S-cis-bifenthrin [35]. The endocrine-disrupting effects of chloroacetamides in fish are related to immunotoxicity and exhibit enantioselective toxicity [27]. Embryonic exposure to permethrin, one of the most frequently used synthetic pyrethroids, until 72 h post-fertilization (hpf) causes cis-permethrin accumulation in zebrafish larvae and results in developmental inhibition by disrupting endocrine signaling at environmentally relevant concentrations [29,45].

In addition to enantioselectivity, toxicity can also be affected by environmental conditions such as temperature and salinity. Temperature variations caused by climate change can have profound implications for pesticide toxicity in aquatic ecosystems and environmental stress in subsequent generations. For example, the association between pesticides and temperature showed negative effects on the ability of fish to detect and escape from contaminated environments, suggesting that temperature influences the ability of an organism to efficiently respond to stress [66]. In parallel with the 0.5-degree temperature increase applied to the parents with glyphosate exposure, a lower survival rate, delay in hatching, increased body malformations, lower blood flow and heart rate, more dark/light locomotor activity, and increased thigmotaxis were detected in the offspring [67]. Although temperature showed no major effects on the accumulation of bifenthrin in zebrafish embryos, an increase in temperature could enhance the toxic effect on the metabolism and physiological thyroid function in zebrafish embryos by regulating related gene expression, including crh, tshβ, nis, tg, pax8, dio1, dio2, ugt1ab, trα, and trβ [35]. Salinity affects the octanol-water partition coefficient of organic contaminants in aquatic ecosystems [68]. From a biological perspective, an organism’s physiology and enzyme metabolism are altered at different salinities, with implications for pesticide metabolism and toxicity. High salinity increases the enrichment of cis-bifenthrin, enhances the influence of cis-bifenthrin on metabolism and thyroid-related gene expression, interferes with the normal physiological functions of metabolism and the thyroid, shows a synergistic effect, and enhances its aquatic toxic effect on zebrafish embryos [35].

In addition, metabolites of different pesticides may pose potential threats. In an investigation on the bioaccumulation, metabolic profile, and toxicity of pyriproxyfen and its metabolites in zebrafish, 16 pyriproxyfen metabolites were identified [69]. Among all the metabolites, 4′-OH-pyriproxyfen was found to be two-fold more toxic to zebrafish than pyriproxyfen [68]. Regarding thyroid dysfunction effects, even exposure to the metabolite 3-phenoxybenzoic acid strongly increases the expression of HPT axis genes in zebrafish larvae and in TRα mRNA levels [21]. The alteration of gene expression of TRβ, Dio2, and TSHβ was induced by β-cypermethrin, while the three metabolites 3-phenoxybenzoic alcohol, 3-phenoxybenzaldehyde, and 3-phenoxybenzoic acid inhibited Dio2 and TSHβ mRNA levels [21]. Although a single dose of metolachlor has no notable effect on thyroid metabolism disruption-associated genes in zebrafish larvae, environmentally relevant concentrations of the pesticide mixture, S-metolachlor, and its two metabolites, metolachlor oxanilic acid and metolachlor ethanesulfonic acid, showed considerable differences [70].

#### 4.1.2. Importance of Exposure-Window Periods and Transgenerational Effects

Pyriproxyfen is a juvenile hormone analog insecticide used worldwide [71,72], indicating the importance of utilizing the drug-sensitive period of pests. Therefore, it is important to understand the exposure-timing effect during development. For example, the critical window for metabolism in zebrafish larvae coincides with hatching time, which may represent an especially vulnerable developmental period [73]. During early development, zebrafish embryos are more sensitive to silver when experiments are initiated at the one-cell stage, but pre-exposure does not influence the outcome of subsequent exposure [74]. Ten to twenty-one days after fertilization is a developmentally sensitive period for zebrafish to transition from larvae to juveniles, during which thyroid hormones play an important role. Aocyclotin, metalaxyl, fipronil, and λ-cyhalothrin markedly inhibit embryo hatching and survival rates indicating direct transgenerational toxicity [22,32,36,38,45]. Acetochlor can disrupt the early development of zebrafish larvae, depending on exposure dose and time [75].

Ecotoxicological studies have shown an association between pesticide pollution and transgenerational toxicity in aquatic organisms because many pesticides can be metabolized and transferred to offspring as new toxicants [32]. Transgenerational effects occur when the physical, developmental, and hormonal characteristics of children are affected by changes in the environment experienced by their parents prior to fertilization [76]. The adverse consequences of parental exposure are transmitted across generations from exposed males and females to their unexposed offspring, affecting the development of first-generation (F1) offspring in a manner that is gender-specific [77]. Cypermethrin accumulates in parental zebrafish and affects the hatching rate, heart rate, and development of swim bladders in F1 generations even when the concentration of cypermethrin is below the limit of quantification in the offspring [78]. Environmental exposure also increases the malformation rate of offspring exposed to maternal pesticides. The rate of malformation (spinal curvature) in offspring notably increases after parental exposure to pentachlorophenol [55]. Cis-bifenthrin induces growth inhibition and neurotoxicity in zebrafish larvae, possibly mediated by developmental interference in embryos related to disrupted fatty acid composition [79]. Transgenerational effects could also be indicated by increased thyroid hormones in both eggs (maternal source) and in developed larvae (newly synthesized), as well as disrupted transcriptional profiles of key genes in the HPT axis [80]. Parental exposure has direct effects on offspring thyroid and TH signals, including the promotion or reduction of T3 and/or T4 levels [31,32], as well as the upregulation of Dio1, Dio2, and Dio3b in F1 fish [32,42]. The potential pesticide risk to offspring development and the lasting thyroid-disrupting effects should always be taken seriously because of the importance of offspring development and population reproduction.

### 4.2. Insecticide and Herbicide Mechanisms on Thyroid and Related Dysfunction

#### 4.2.1. On Thyroid System

**Figure 2:**
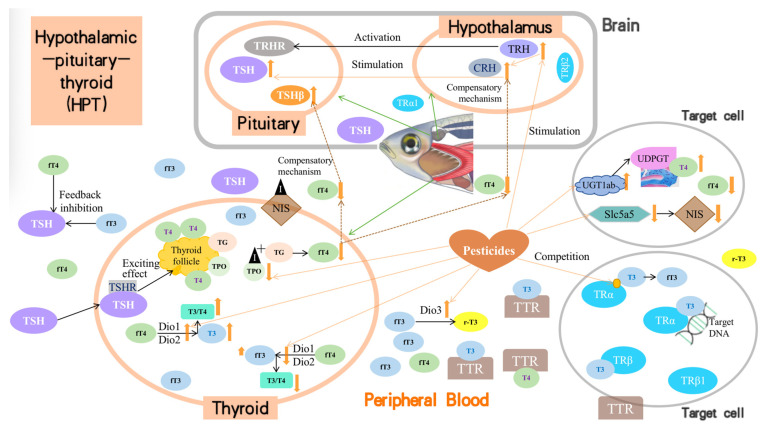
Mechanisms of insecticides/herbicides on HPT-related zebrafish dysfunction.

Insecticides and herbicides may act on different endocrine axes, such as the HPG, HPT, and HPA, to disrupt the endocrine system. Under normal conditions in zebrafish, the hypothalamus first secretes thyroid CRH, which then regulates TSH secretion by the pituitary gland, thyroid synthesis, and secretion of thyroid hormones. Most pesticides directly interfere with the levels and functioning of TH (T3 and T4) in zebrafish, thereby interfering with thyroid function. Generally, all the reviewed pesticides cause thyroid dysfunction in the following ways (Figure 2). (1) They stimulate TH synthesis through upregulation of the mRNA expression of critical genes, such as TRH and CRH in the hypothalamus or TSH in the pituitary, although this upregulation is not steady in the combined toxicity mode. (2) They promote or inhibit critical enzymes that control TH synthesis or metabolism in the thyroid or blood, such as Dio1, Dio2, TPO, TG, and T3-regulated cpt1, igf3, and igfbp1a. (3) They alter the expression of other critical proteins that are involved in the transportation, degradation, or reception of THs, including Dio3, TTR, NIS, UGT1ab, TRα, and TRβ, amongst others. (4) They affect genes known to play an essential role in thyroid development, including the transcription factors Nkx2.1 and Pax8, among others. (5) The regulation of parameters (2), (3), and (4) passed on the effects of the transcription of genes involved in thyroid function. Meanwhile, based on the competition, compensatory, and even feedback inhibition mechanisms, altered levels of T3 and/or T4 could increase the response of the entire HPT axis of zebrafish and reduce the adverse effects caused by the target pollutants.

Comparing the effective thyroid dysfunctional concentration of different insecticides in Table 1, pyrethroids show higher toxicity at the mean concentration level of 0.1 µg/L for bifenthrin, permethrin, and λ-cyhalothrin but the highest for fenpropathrin at 0.015 µg/L. For other insecticides, the sensitivity sequence of the zebrafish thyroid system was generally azocyclotin, ethiprole, fenvalerate, triazophos, chlorpyrifos, carbofuran, β-cypermethrin, pyriproxyfen, and imidacloprid. The effective concentration of other insecticides for inducing thyroid dysfunction in zebrafish should be at least 1 mg/L. For herbicides (Table 2), the zebrafish thyroid system is more sensitive to atrazine, with an effective concentration of 0.3 µg/L, followed by pentachlorophenol, acetochlor, butachlor, ioxynil, pretilachlor, and metolachlor at the level µg/L. In addition to the differences in the toxicity of the thyroid interference effects, there is also a complex simulated or competitive relationship between pesticides and thyroid hormones. Increased concentrations of acetochlor produce a strong growth-promoting effect, similar to that of T3 [58]. A possible competitive relationship exists between azocyclotin and T3 in vivo [22]. Chlorpyrifos shows weaker binding ability to the TRβ compared to T3, and the disturbance of thyroid signaling in zebrafish might arise from the developmental neurotoxicity induced by chlorpyrifos [25].

#### 4.2.2. Multi-Organ and Organ-Dependent Effects of Pesticides

THs play important roles in the regulation of vertebrate development, growth, and reproduction in vertebrates [81]. T3 levels define the hypothyroid/hyperthyroid status of each organ. Thyroid diseases are often associated with pathologies, such as obesity and non-alcoholic fatty liver disease, among others [82]. Simazine residues in natural waters would cause necrosis in the kidney and liver and hepatic steatosis in carp from contaminated areas [83]. In adult zebrafish, sex-dependent damage of hepatic T3-level signaling was associated with liver steatosis, which was more pronounced in females, with sex-dependent alteration of transcripts codifying the key enzymes involved in ‘de novo lipogenesis’ and β-oxidation [31].

Sex steroids are known to regulate vertebrate sexual development, reproduction, and behavior, whereas THs regulate metabolism, growth, and development. Cross-talk between steroid hormones and thyroid axes has been observed. Reproductive processes in fish are regulated by coordinated interactions between steroid hormones along the HPG axis and the steroidogenesis of gonadal tissues [84]. Theoretically, environmental contaminants that affect the expression of steroidogenic genes and the concentration of hormones in this axis could affect the function of the endocrine system and possibly reproduction in fish [85]. Thiamethoxam has different interactions with ERα, AR, and TRα and simultaneously induces a plasma T4 decrease and histological damage in the liver and delayed gonadal development in both genders of Chinese rare minnow (*Gobiocypris rarus*) [86]. THs regulate sex steroid synthesis and action in both the brain and gonads, which are important for gonadal development and brain sexual differentiation and have been studied in many species [87]. THs control the migration and differentiation of neurons, oligodendrocytes, astrocytes, and microglia in the brain [88,89], and both androgens and estrogens play important roles in brain development [90]. Although sex steroid-related genes are expressed in the brain during development and adulthood in vertebrates, no consistent results regarding the sexually dimorphic expression of sex steroid-related genes during brain development have been confirmed to date.

Thyroid disruptors, which cause molecular changes in the thyroid system, have been reported to impair the visual capacity of *D. rerio* larvae [91]. Eye development in zebrafish occurs between 12 and 72 hpf in early life stages (embryonic and larval) and has become a strong research tool in developmental toxicity investigations [92]. The biggest concern is that, from this life stage, larval and adult retinas are anatomically and functionally similar to humans [93,94]. As the most distinctive herbicide, 2,4-D damages the structure of the larval retina based only on early exposure [95], which has not been commonly found in pesticides or herbicides but more in fungicides [96,97]. Larval retina is regularly shaped, intact, and comprises six typical laminae, while in 2,4-D-exposed zebrafish, different morphological alterations were observed in the retina, including widespread empty areas in the ganglion cell layer, increased thickness and/or partial detachment of the retinal pigment epithelium from the photoreceptor cell layer, and partial detachment of the photoreceptor cell layer from the outer plexiform layer [95]. Therefore, it is proposed that a 2,4-D-mechanistic effect may occur through its effects on axonal growth via the TH-dependent pathway [98].

Heart function dynamically changes thyroid morphology and function; hence, it is likely that the observed cardiac effects of the target pollutants are the source of altered thyroid status in fish [99]. Perfluorononanoic acid exerts severe cardiotoxic effects and, in addition to reproductive toxicity and hepatotoxicity, disrupts thyroid function and damages embryonic development in zebrafish [100]. Chronic exposure to ioxynil induces ventricle deformation, a notable volume increase in the zebrafish heart, thyroid follicle morphology changes [99], and the inhibition of thyroid gland development, strongly associated with altered heart morphology [63].

## 5. Conclusions

The effective concentration of insecticides on thyroid function damage in zebrafish was lower than that of the investigated herbicides, indicating that the overall toxicity of insecticides on the thyroid of zebrafish was greater than that of herbicides. Fenpropathrin and atrazine are the most serious thyroid function disruptors among all the investigated pesticides, with effective concentrations of 0.015 µg/L and 0.3 µg/L, respectively. Fenpropathrin, bifenthrin, permethrin, and acetochlor showed more sophisticated single and/or combined toxicity effects due to the variety of morphological characteristics of chiral pesticides, even with sex differences. Besides alteration of TH levels and expression of genes related to the HPT axis, complicated effects on embryo/larvae growth, development of the skeleton, heart, and retina, changes to the antioxidant system, cellular apoptosis, reproductive hormone expression, inflammation system, and even offspring survival could all be observed.

The sensitivity and significance of indicators, including T3 and T4 levels and the expression of genes related to the HPT axis to determine the thyroid dysfunction of zebrafish, can be seen in the obtained results. The application of indicators including TSH, free T3, free T4, T3, T4, TG antibodies, TPO antibodies, and TR antibodies in human medical tests as a standardized procedure has proved the reliability of these sensitive indicators for diagnosis of human thyroid disease/thyroid dysfunction disease. In light of this, zebrafish could be further investigated for screening indicators for thyroid dysfunction-related diseases, including obesity, non-alcoholic fatty liver disease, gonad development problems, abnormal brain sexual differentiation, retinal structure damage, and heart morphology alterations, for human clinical medicine. By summarizing the induction potential and main mechanism of thyroid dysfunction of target pesticides, zebrafish mutants can be constructed in the high-throughput screening of water pollutants, not only pesticides, which can be established in future studies. Moreover, important tools for early warnings of water environmental pollution risks and basin-combined pollution control can be screened, and control over the production and use of pesticides in China can be strengthened.

## Figures and Tables

**Table 2:** Parameters on thyroid disturbance effects of herbicides on zebrafish [49,50,51,52,53,54,55,56,57,58,59,60,61,62,63,64,65].

Herbicide Type	Name	MC.	EC.	Time	Life Stage	Refs.
(1) Amides	Acetochlor	30 μM	2 μM	72 h	Embryo, larvae	[27]
		300 µg/L	3 µg/L	14 d	Embryo, larvae	[57]
		300 µg/L	60 µg/L	14 d	Embryo, larvae	[65]
		300 µg/L	3 µg/L	14 d	Embryo, larvae	[58]
		30 μg/L	10 μg/L	14 d	Embryo, larvae	[28]
		50 μg/L	1 μg/L	120 hpf	Embryo, larvae	[59]
		0.012 µM	0.004 µM	14 d	Embryo, larvae	[58]
	Butachlor	100 µg/L	50 µg/L	30 d	Adult	[49]
		100 µg/L	50 µg/L	30 d	Adult	[50]
		20 μM	4 μM	84 hpf	Embryo, larvae	[27]
		32 µg/L	6.4 µg/L	10 d	Embryo, larvae	[51]
	Metolachlor	100 μg/L	100 μg/L	120 h	Embryo	[62]
	Pretilachlor	200 µg/L	50 µg/L	96 h	Embryo	[52]
(2) Organophosphorus	Glyphosate	35 mg/L	7 mg/L	120 hpf	Embryo, larvae	[54]
(3) Phenols	Pentachlorophenol	27 µg/L	1 µg/L	70 d	Adult	[55]
(4) Phenoxy carboxylic acid	2,4-D	1.5 mM	24.9 µM	5 dpf	Larvae	[56]
(5) Sulfonylurea	Ioxynil	1 µM	0.1 µM	36 h	Eggs, embryos	[63]
(6) Triazene	Atrazine	30 µg/L	0.3 µg/L	7 dpf	Embryo	[64]
		100 μg/L	1 μg/L	3 d	Larvae	[53]

**Table 3:** Parameters on thyroid disturbance effects of combined insecticides and herbicides in zebrafish.

Pesticide Type	Name	EC.	MC.	Time	Life Stage	Refs.
Insecticide	Bifenthrin	0.0024 µM	0.0072 µM	14 d	Embryo, larvae	[28,59]
Herbicide	Acetochlor	0.004 µM	0.012 µM			
Insecticide	Triazophos	0.002 mg/L	0.032 mg/L	96 h	Whole life	[23]
Insecticide	Imidacloprid	0.38 mg/L	6.08 mg/L			
Insecticide	Chlorpyrifos	30 nM	300 nM	180 dpf	F1 larvae, adult, F2 embryo, larvae	[31]
Insecticide	Ethylene thiourea	100 μM	100 μM			
Insecticide	Chlorpyrifos	30 nM	300 nM	180 dpf	Larvae, adult	[26]
Insecticide	Ethylene thiourea	100 μM	100 μM			

## Data Availability

The data presented in this study are available on request from the corresponding author.

## Supplementary Materials

The following supporting information can be downloaded at: https://www.mdpi.com/article/10.3390/toxics12080570/s1, Table S1: Effects of insecticide on thyroid and other system disturbances in zebrafish; Table S2: Effects of herbicides on thyroid and other system disturbances in zebrafish.
