# Supplementary material for: Effects of Insecticide and Herbicides on Thyroid Disturbances in Zebrafish
Source: Toxics. 2024 Aug 3;12(8):570. doi: 10.3390/toxics12080570 (PMC11358992; doi:10.3390/toxics12080570)
Supplement: Supplementary file 1 [file toxics-12-00570-s001.zip › toxics-3115791-supplementary.pdf]

Table S1. Effects of insecticide on thyroid and other system disturbances in zebrafish.

Index	Azocyclotin	Metalaxyl	Carbofuran	Pyriproxyfen	Imidacloprid	Chlorpyrifos	Triazophos	Fipronil	Ethiprole	Flufiprole	Bifenthrin	Fenpropathrin	Fenvalerate	Permethrin	β-cypermethrin	λ-cyhalothrin	Ethylene thiourea
TH level	T3, T3/T4+ [22]		T4+ [30]		T3+, T4- [23]	T3+ [24, 25, 26]	T3+, T4- [23]	T3, T4 F0, F1- [32]			T3, T3/T4+; T4- [27]; T3, T4+ [28]			T3, T3/T4, T4+ [27, 29]		T3, T3/T4, T4+ [27]	T3, T4+ F1 [31]
HPT axis related	Dio2- [22]	Nis+R-; TPO-R-; Dio2+rac-; dio1-, dio2+ R- [38]		TSHβ-; dio1, dio2+; TRα- [34]	TSH-; dio1, dio2- [23]	TSH+; Dio1, Dio2+ [24]; tshbα+; TPO+; TTR+ [25]; Dio3b [42]; Ugt1ab+, crhβ+ [25]; CRH+ [24]; TG-; TRα+ [24]; thrβ+; slc5a5+ [25]	TSH-; dio1, dio2-; TRβ+ [23]	TSHβ+ F0; NIS+F0, F1; TPO+ F0, F1; TTR+F0; Dio1, Dio2+ F0, F1; Utg1ab+ F0; CRH+ F0; TG+ F0; TRα+ F0; TRα- F1; Nkx2.1+ F0, F1 [32]	TSHβ+ S-; Dio1- S-; TRβ- S- [39, 40]	TSHβ+ S-; Dio1- S-; TRβ- S- [39, 40]	TSHβ+ [27, 28, 35]; Nis+ [35]; TTR+ [27, 35]; Dio2+ [27]; Dio1+ [35]; Dio2+ [28, 35]; Ugt1ab+ [27, 35]; UTG1A1+ 1R-cis0 [35]; CRH+ [27, 28, 35]; TG+; TRβ+ [35]; TRα+ [27, 28, 35]; Pax8 + [27]; CYP1A+ 1S-cis-, CYP3A+, Pax8, Nkx2.1+ [35]	NIS+ Rac, S-Fen- juvenile; TSHβ- Rac; R-Fen juvenile; TPO+ embryo S-Fen; TPO- larvae Rac; TTR+ S-Fen larvae; -R-Fen juvenile; Dio1- embryo R-Fen, larvae Rac; Dio2- juvenile; UTG1ab+ embryo S-Fen; -larvae, juvenile Rac, R-Fen; CRH- Rac, S-Fen juvenile; TG- Rac, S-Fen embryo, juvenile; + R-Fen larvae; TRα- larvae, juvenile S-Fen, Rac; Nkx2.1- Rac, R-Fen; Pax 8- S-Fen juvenile [41]	TRα, TRβ- embryo [36, 45]	NIS- [36, 45]; TSHβ+ [21, 27, 29, 36]; TPO+ [36]; TTR+; Dio1, Dio2+ [27, 29]; Dio1, Dio2+ [36]; Dio2+ [21]; CRH+ [27, 29, 36]; TG+ [36]; TRα+; TRβ- [27, 29]; TRα, TRβ, TRH, TRHR+ [36]; TRα, TRβ- embryo [36, 45]; TRα+ [21]; Pax8, TRO+ [27, 29]	TSHβ+; Dio2+; TRα, TRβ+ [21]	Nis+ [35, 36, 45]; TSHβ+ [35]; TSHR, TSHβ+ [36]; TPO- [36]; TTR+ [27, 35]; Dio1, Dio2 + [27]; Dio1, Dio2+ [35]; Dio1, Dio2+ [36]; Ugt1ab+ [35]; CRH+ [35, 36]; TG+ [35, 36]; TRα+ [27]; TRα, TRβ+ [35]; TRα, TRβ-, TRH, TRHR+ [36]; TRα, TRβ- embryo [36, 45]; Pax8+ [27]; Pax8, Nkx2.1+ [35]	TSHβ+ F1; TPO+; Dio3b+ F1 [31]; Dio2+ [26]; Ugt1ab+ F1; TG+; TRH+; igfbp1a+ [31]; igf3, igfbp1a+ [26]
Offspring production	Hatching rate- [22]	Hatching rate-, survival rate- [38]						F1 Hatching rate-, survival rate- [32]								Hatching rate- [36, 45]	
Embryotoxicity	Heart beat- [22]	Yolk sac edema; Pericardial edema [38]									Heart beat- [35]	Yolk sac edema; Pericardial edema [41]		Pericardial edema [21]	Pericardial edema [21]	Heart beat- [35]	
Growth	Body weight- [22]			Body length, body							Death rate+ [28, 35]		Mortality+ [36, 45]	Body weight- [27, 29];		Body weight- [27]; Death	

			weight, growth hormone- [34]										Mortality+ [36, 45]		rate+ [35]; Mortality+ [36, 45]
Skeleton and movement	Crooked body; short tail [38]						Malformation rate+; Swim speed- F1 [32]			Curved spine [35]	crooked body [41]				Curved spine [35]; malformation [36, 45]
Sexual hormone	Vtg1, cyp19a, cyp19b-R- [38]				ERα, ERβ1, ERβ2, VTG1, VTG2+ [24]							AR-, ER1, ER2α+ [36, 45]	AR- ER1, ER2α, ER2β+ [36, 45]		AR-, ER1+, ER2α, ER2β- [36, 45]
Oxidative damage				Mn-sod, gpx-; cat+ [23]		Mn-sod, gpx-; Cu/Zn-sod+ [23]	EROD, CTP1A+ F0 [32]			CAT+ [28]					
Apoptosis				bcl-2, ucp-2, bax, p53+; cas3, cas9- [23]		bcl-2, ucp-2, bax+; cas3, cas9- [23]									
Immunity				CXCL-CIC, CC-chem, IL-1β, IL-8- [23]		CXCL-CIC, CC-chem, IL-1β, IL-8- [23]							IL-1β, IL-8, CXCL-Clc, CC-chem [21]	IL-1β, IL-8, CXCL-Clc, CC-chem [21]	

Table S2 Effects of herbicides on thyroid and other system disturbances in zebrafish.

Index	Acetochlor	Butachlor	Metolachlor	Pretilachlor	Glyphosate	Pentachlorophenol	2,4-D	Ioxynil	Atrazine
TH level	T3-, T4+ [57]; T3, T4+ [28, 59]; T3, T3/T4+, T4- [65]	T3, T4+ [49, 50, 51]		T3, T4+ [52]	T3/T4- [54]	T3+, T4- male [55]	T4- [56]		T4+ [53]
HPT axis related	TSHβ+ (-)-R, (+)-S [27]; tsh- [75]; tsh+; tpo+ [57]; TTR- [57, 75]; Dio2+ (-)-R, (+)-S [27]; Dio2+ [28, 59, 65]; Utg1ab- [75]; Ugt1- [57]; crh- [75]; TRα, TRβ+ (-)-R, (+)-S [27]; TRα, TRβ- [75]; trα+ [57]; TRα- (-)-R, + (+)-S; TRβ+ (+)-S, - (-)-R, Rac [65]; slc5a5- [75]; slc5a+; dl- [57]	TSH+ [49]; tpo+ [51]; TTR+ [49, 51]; Dio1+ [49]; Dio2+ [51]; Utg1ab-; CRH+ [49]; tg+ [51]); TRα+ [49]; trβ+ [51]; Slc5a5+ [49]	Dio2 +; thrβ+ [70]	Dio2+; TRα+ [52]	TSHβ+; TTR+; TRα, TRβ+ [54]	TSHβ- brain; TTR+ female liver; - male liver; Doi1- liver; Dio2+ female liver; Dio2- male liver; Utg1ab+ liver; sult1st5- female liver; sult1st5+ male liver [55]		Tg-; Nkx2.1a- [63]	THRA- [64]
Offspring production	Mortality+ [27, 28]; hatching rate- [57]	Hatching rate- [27]; survival rate- [51]			Hatching rate+ [54]				
Embryotoxicity	Body weight+ [57]	Body weight+ [49]			Body length- [54]				
Growth	Coagulation [27, 65]; Yolk sac edema [27, 57, 65, 75] Pericardial edema [27, 65]	Yolk sac edema; Pericardial edema [27]			Swim bladder deficiency, spinal curvature; pericardial edema, heart rate+ [54]				
Skeleton and movement	Crooked body [27, 65]; notochord deformation [57, 75]	Caudal vertebra deformation [51]			Tail malformation, short tail, head malformation. spinal curvature [54]				
Sexual hormone		Vtg1+ [27]		VTG1, CYP19a, CYP19b, ERα+; ERβ1- [52]					

Oxidative damage	MDA+ [57]; CAT+ [28]	SOD, GST, activity, GSH- [49]		Cu/Zn-SOD, GPX+ [52]	MDA+ [54]
Apoptosis			P53+ [70]	P53, Bbc3, Cas 3, Cas9+ ([52])	Cas8, Cas9, p53+ [54]
Immunity	CXCL-CIC, CC-chem, IL-1β, IL-8+ [27]	CC-chem, IL-1β, IL-8+ [27]		CXCL-CIC, IL-1β, IL-8- [52]	iNOS, TNF-α, IL-1β+ [54]
